# Supervillin Is a Component of the Hair Cell’s Cuticular Plate and the Head Plates of Organ of Corti Supporting Cells

**DOI:** 10.1371/journal.pone.0158349

**Published:** 2016-07-14

**Authors:** Lana M. Pollock, Nilay Gupta, Xi Chen, Elizabeth J. Luna, Brian M. McDermott

**Affiliations:** 1 Department of Otolaryngology–Head and Neck Surgery, Case Western Reserve University, Cleveland, Ohio, 44106, United States of America; 2 Department of Genetics and Genome Sciences, Case Western Reserve University, Cleveland, Ohio, 44106, United States of America; 3 Department of Biology, Case Western Reserve University, Cleveland, Ohio, 44106, United States of America; 4 Department of Neurosciences, Case Western Reserve University, Cleveland, Ohio, 44016, United States of America; 5 Department of Cell and Developmental Biology, University of Massachusetts Medical School, Worcester, Massachusetts, 01605, United States of America; Harvard University, UNITED STATES

## Abstract

The organ of Corti has evolved a panoply of cells with extraordinary morphological specializations to harness, direct, and transduce mechanical energy into electrical signals. Among the cells with prominent apical specializations are hair cells and nearby supporting cells. At the apical surface of each hair cell is a mechanosensitive hair bundle of filamentous actin (F-actin)-based stereocilia, which insert rootlets into the F-actin meshwork of the underlying cuticular plate, a rigid organelle considered to hold the stereocilia in place. Little is known about the protein composition and development of the cuticular plate or the apicolateral specializations of organ of Corti supporting cells. We show that supervillin, an F-actin cross-linking protein, localizes to cuticular plates in hair cells of the mouse cochlea and vestibule and zebrafish sensory epithelia. Moreover, supervillin localizes near the apicolateral margins within the head plates of Deiters’ cells and outer pillar cells, and proximal to the apicolateral margins of inner phalangeal cells, adjacent to the junctions with neighboring hair cells. Overall, supervillin localization suggests this protein may shape the surface structure of the organ of Corti.

## Introduction

The hair cells of the inner ear are crucial to detection of stimuli associated with hearing and balance. Protruding from the apical surface of each hair cell is an array of F-actin-based stereocilia, forming the mechanosensitive hair bundle [1]. Each stereocilium tapers at its base, inserting as a densely-packed rootlet into the underlying cuticular plate (CP), a stiff actin gel hypothesized to anchor the stereocilia to hold them upright [2, 3]. The CP may also be involved in mechanical adaptation following stereocilia deflection and control vesicular transport [4]. However, the precise roles of the CP in hair cell development and maintenance have been difficult to establish, in part due to lack of knowledge of the protein composition of this unique structure.

*In vitro*, gel-like meshworks of F-actin form in the presence of actin-bundling proteins, which organize and cross-link neighboring filaments [5]. The mechanical properties of actin gels depend on the specific type and concentration of actin-bundling proteins involved. Within the CP meshwork, cross-linkers connect adjacent actin filaments, and other linkers connect stereociliary rootlets to the CP or the CP to the overlying plasma membrane [6–8]. However, the identity of these various linkers remains unknown [9].

In the mouse organ of Corti (S1 Fig), apical regions of hair cells and neighboring non-sensory supporting cells, including Deiters’, pillar, and inner phalangeal cells, are tightly connected by junctional proteins, forming the reticular lamina [10]. The apical region of each Deiters’ and pillar cell expands outward, forming a head plate filling the space between neighboring hair cells [11]. The reticular lamina assists in maintaining organ of Corti tissue integrity and provides a physical barrier separating the differing ionic contents of the cochlea, endolymph and perilymph [12–14]. Circumferential F-actin belts in both hair cells and supporting cells are associated with the cell junctions, and the supporting cell belts are thought to be involved in myosin-dependent scar formation upon loss of nearby hair cells [15–17].

**Fig 1:**
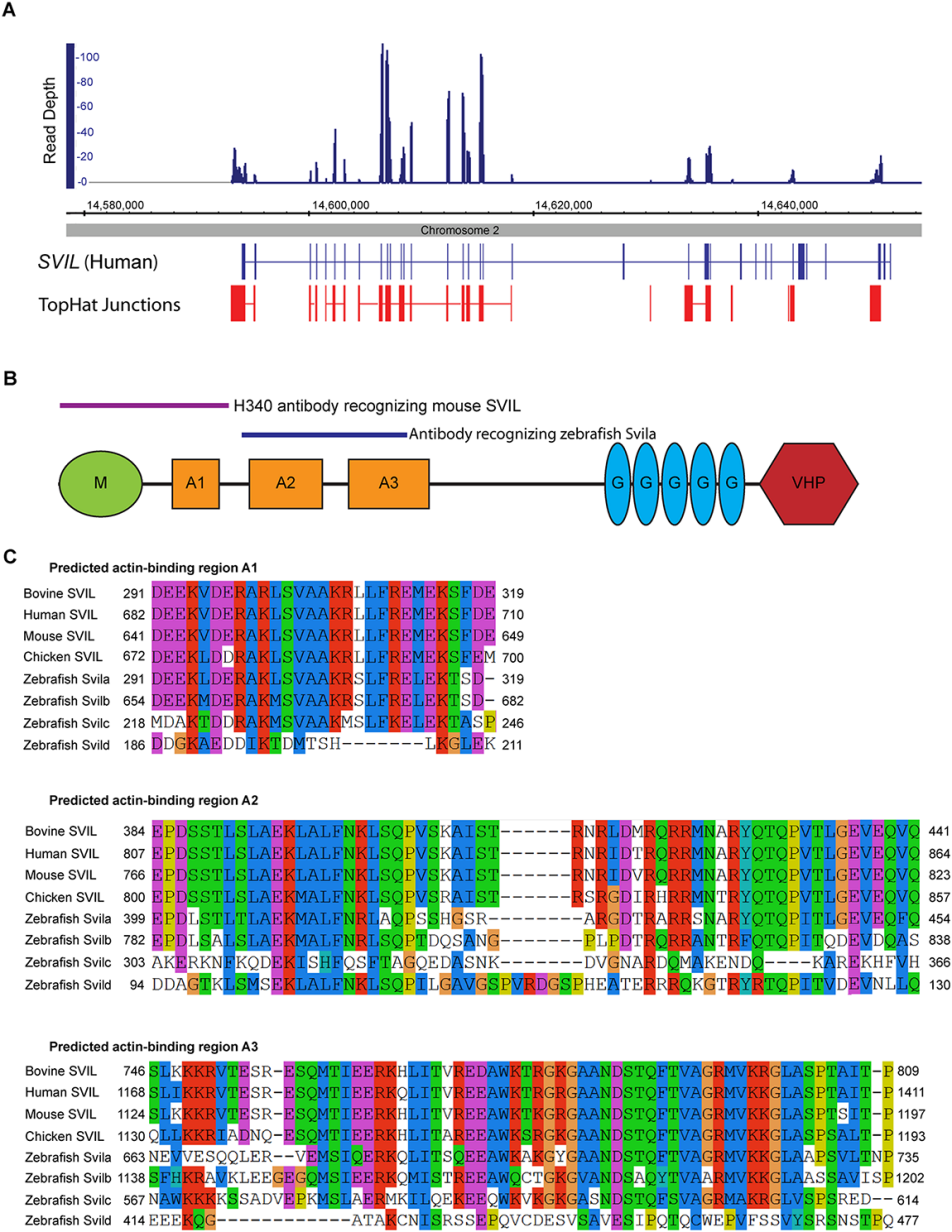
The gene encoding supervillin is expressed in chicken hair cells. **(A)** Detection of *SVIL* mRNA in chicken hair cells by RNA-seq. Depth of reads aligned to the chicken genome, with TopHat-predicted splice junctions (red) and exons of human *SVIL* aligned to the chicken genome (blue). **(B)** Major functional domains of supervillin: M, myosin II-binding region; A1-A3, actin-binding regions 1–3; G, gelsolin repeats; and VHP, villin headpiece. Purple line indicates region of mouse SVIL recognized by the H340 antibody (Oh et al., 2003), and the blue line indicates the region of zebrafish Svila recognized by novel antiserum. **(C)** Alignment of vertebrate supervillin protein sequences using Clustal/Jalview and default parameters. The regions of bovine supervillin shown to bind the myosin II heavy chain and F-actin [19] are displayed.

Supervillin, a ~205-kD villin/gelsolin superfamily protein [18], directly binds and cross-links F-actin [19, 20]. Supervillin also regulates myosin II contractility subjacent to plasma membranes [21–23]. Towards the protein C-terminus is a series of gelsolin homology domains and a villin headpiece [18] (Fig 1B), which surprisingly do not have affinity for F-actin in cell culture models [19, 20]. Instead, the supervillin N-terminal region binds the myosin II heavy chain at one site and F-actin through three other nearby sequences [20, 23]. In confluent kidney epithelial cells, supervillin co-localizes with E-cadherin at sites of lateral cell-cell contact, where it is thought to be involved in cytoskeletal assembly or stabilization at adherens junctions [18]. Here, we identify supervillin as a potential linker between actin filaments in the developing CP and within cochlear supporting cells.

## Materials and Methods

### Animals

Zebrafish (*Danio rerio*) strains *Gt(macf1a–citrine)*^*ct68a*/+^ [24], GFP-fascin 2b [25, 26], and wild-type strain *Tübingen* were used as well as *White Leghorn* chickens (*Gallus gallus*) and *FVB/NJ* mice (*Mus musculus*). All animals were kept with the approval of the Case Western Reserve University Institutional Animal Care and Use Committee (protocol numbers 2012–0187, zebrafish, 2013–0031, mouse, and 2011–0161, chicken). Zebrafish were euthanized by chilling at 4 degrees Celsius, and mice and chickens were euthanized by carbon dioxide inhalation followed by cervical dislocation. All mouse, zebrafish, and chicken experimental protocols were approved by the Institutional Animal Care and Use Committee at Case Western Reserve University.

### RNA-seq

Isolation of chicken hair cells and RNA-seq were performed [27]. Potential orthologs of transcripts that did not align to an annotated chicken gene were identified by comparison with the Swiss-Prot database using NCBI BLAST. Exons of human *SVIL* aligned to the chicken genome are displayed in Fig 1A using the Integrated Genome Browser through Galaxy [28].

### Reverse transcription-polymerase chain reaction (RT-PCR)

Isolation of hair cells and macular tissue from adult mice and zebrafish and generation of cDNA has been described [27, 29]. Primer pairs used are in S1 Table.

### Whole-mount mRNA *in situ* hybridization

Seven-dpf zebrafish embryos were used to synthesize cDNA [29]. Fragments of *svila* cDNA and *svilc* cDNA were amplified by PCR using primers svila_insitu_fwd 5ʹ-ACAAACAGATGGAGAGCACACAAC-3ʹ, svila_insitu_rev 5ʹ- ACATGAGTACACGGAACAAAGACTG-3ʹ, svilc_insitu_fwd 5ʹ- AACGGATCGCTCGCTACAAAG-3ʹ, and svilc_insitu_rev 5ʹ- ACTTCATCCACTGTGATGGG-3ʹ. Resulting products were cloned into pCRII vectors (Invitrogen, USA), from which sense and antisense probes were synthesized for *in situ* hybridization [29].

### Immunofluorescence of mouse tissues

Vestibular tissue from mice at P1, P3, and 6 months of age were dissected and then immediately fixed 10 minutes in ice-cold methanol. For labeling of cochlear hair cells, the organ of Corti was removed from mice of different ages, cultured overnight [30], and then fixed 10 minutes in ice-cold methanol.

Following fixation, vestibular or cochlear tissue was washed in phosphate-buffered saline (PBS), blocked in 2% bovine serum albumin (BSA) for 1 hour, and then incubated with primary antibodies diluted in 2% BSA overnight. Primary antibodies were anti-H340 rabbit polyclonal recognizing SVIL [31], mouse monoclonal anti-actin (1:100, Clone C4, Millipore, Germany), mouse monoclonal anti-acetylated α-tubulin (1:100, 6-11B1, Sigma, USA), mouse monoclonal anti-β-catenin (1:200, BD Transduction Laboratories, USA), and mouse monoclonal anti-ZO-1 (Invitrogen; Cat. #: 339100). Secondary antibodies were Alexa Fluor 488 chicken anti-rabbit IgG (1:200) and Alexa Fluor 546 goat anti-mouse IgG (1:200, Invitrogen, USA). Alexa Fluor 633 phalloidin (1:50, Invitrogen, USA) was used. Tissue mounted in Vectashield (Vector Laboratories, USA) was imaged on a Leica SP2 or SP8 confocal microscope using a 40 × or 63 × objective (Leica Confocal Software, Leica, Germany).

### Preparation of zebrafish Svila antibody

A novel rabbit polyclonal antiserum was generated against amino acids 364–723 of zebrafish Svila. The corresponding *svila* cDNA sequence was amplified by PCR using cDNA from adult zebrafish maculae and primers Zf_svila_antigen_F 5ʹ-AACCCGGGCAAAGCTCCATGGTGAGAGAGCAGGCCAGAG-3ʹ and Zf_svila_antigen_R 5ʹ-AAGAATTCTCATACTCGCTCAAGCTGTTGGCTTTCCACAACTTCATTTCCC-3ʹ. The resulting product was inserted into pCR8/GW/TOPO (Life Technologies, USA), which was subsequently digested with *XmaI* and *EcoRI*. The isolated *svila* fragment was cloned into pGEX-3X, which was used for glutathione S-transferase (GST)-fusion protein expression. The cognate protein was used in immunization and affinity purification (Proteintech, Inc, USA).

### Zebrafish immunofluorescence

Embryos at 4 dpf were fixed in ice-cold Cytoskelfix (Cytoskeleton Inc., USA) for 10 minutes, permeabilized in 1.5% Triton X-100 (Sigma, USA) for 1 hour, then blocked with 5% goat serum overnight. Embryos were incubated with anti-Svila (1:200) and anti-acetylated α-tubulin (6-11B1; Sigma, USA), washed in PBS, then incubated with Alexa Fluor 633 goat anti-rabbit IgG (1:200) and Alexa 546 goat anti-mouse IgG (1:200 Invitrogen, USA). Phalloidin labeling of *Gt(macf1a–citrine)*^*ct68a*/+^ fish was as described previously [27]. Fish were imaged under a 40 × objective on a confocal microscope (Leica, Germany).

### Antisense morpholinos

A morpholino antisense oligonucleotide targeting the translation start site of *svila* was synthesized by Gene Tools, LLC (USA) with the sequence 5ʹ-GTGCAATTCGCTCCTTCCTGTTCAT-3ʹ along with a 5-base pair (bp)-mismatch control oligonucleotide with the sequence 5ʹ-GTGCAATTCGCTCCTTCCTGTTCAT-3ʹ. Zebrafish embryos at the single-cell stage were injected with 150 pg of either the Svila or control morpholinos, and phenotype analysis was conducted at 4 dpf by immunolabeling, as described above, and image analysis using Leica Confocal Software (Leica, Germany). In the hair cells of immunolabeled morphant and control fish, the fluorescence intensity of anti-Svila-associated signal was measured at the CP using the Leica Confocal Software and then compared to the intensity of anti-α-tubulin-associated signal measured immediately below the CP. The average ratio of anti-Svila fluorescence intensity to anti-α-tubulin fluorescence intensity in control and morphant fish is reported ± the standard error of the mean.

## Results and Discussion

### *Supervillin* is expressed in vertebrate hair cells

To identify candidate actin-interacting proteins potentially involved in shaping the unique cytoskeletal structures of hair cells, we examined the transcriptome of manually-isolated chicken hair cells by RNA-seq. *SVIL* mRNA encoding supervillin was highly abundant (Fig 1A), with 4.09 reads per kilobase per million mapped reads (RPKM). This was more abundant than several genes known to be expressed in hair cells, including protocadherin 15 (*PCDH15*) mRNA [32], which had an RPKM value of 2.09. Supervillin binds myosin II and F-actin with regions near the protein N-terminus (M and A1, A2, A3, respectively, in Fig 1B) [19]. All four binding regions [19, 33] are conserved in vertebrates (Fig 1C). Based on this information, we hypothesized supervillin may localize to specific actin-rich organelles in the hair cell, as it localizes to invadopodia in MDA-MB-231 metastatic breast carcinoma cells [34] or contractile rings in dividing HeLa cells [35].

**Fig 2:**
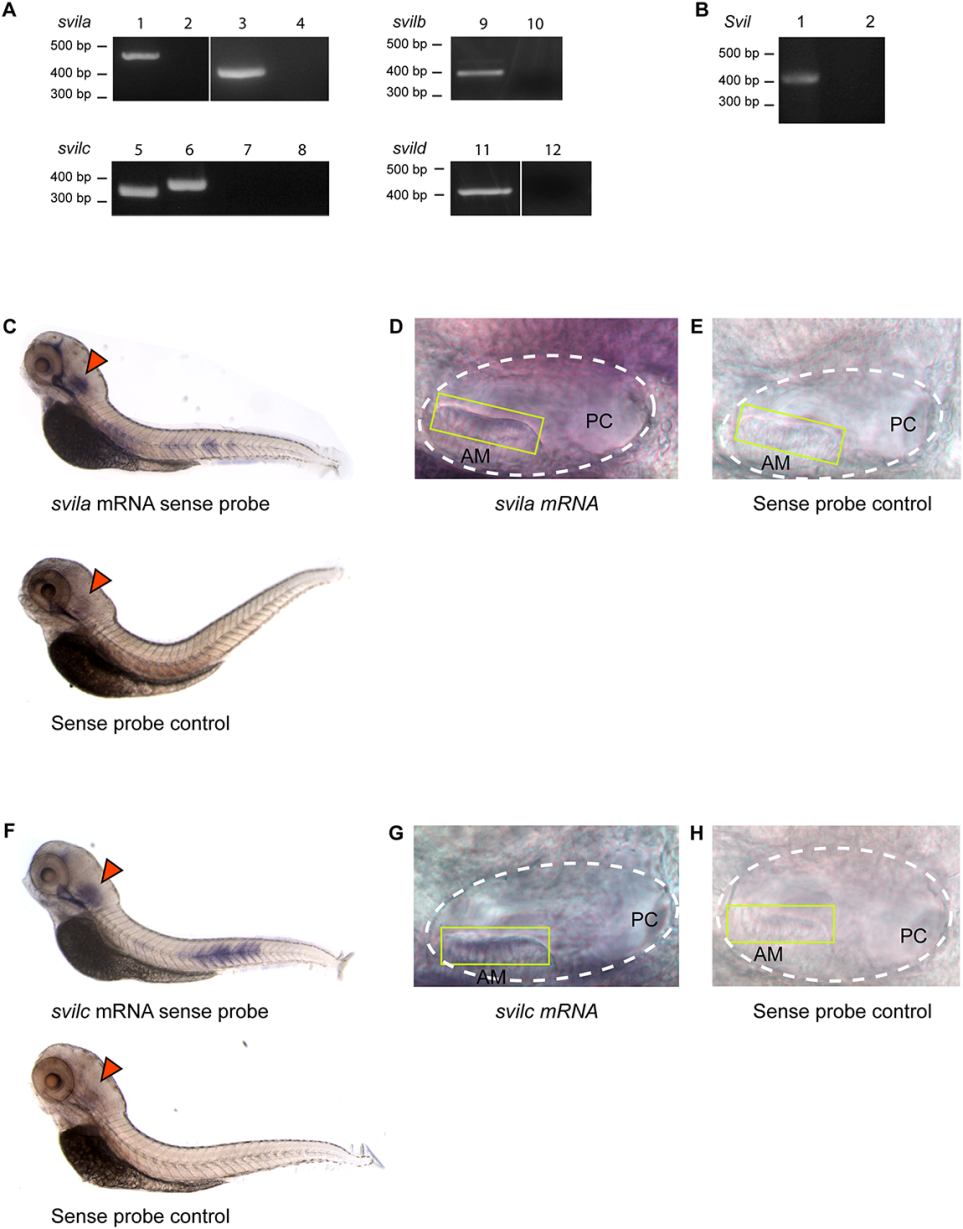
Supervillin is expressed in the mouse and zebrafish ear. **(A)** RT-PCR detection of *svila* and *svilc* mRNAs in zebrafish hair cells using two primer pairs to detect *svila* mRNA [RT plus (lanes 1,3), RT minus (lanes 2,4)] and two primer pairs to detect *svilc* mRNA [RT plus (lane 5,6), RT minus (lane 7,8)]. *Svilb* [RT plus (lane 9), RT minus (lane 10)] and *svild* [RT plus (lane 11), RT minus (lane 12)] mRNAs were detected in zebrafish maculae. **(B)** RT-PCR of *Svil* mRNA from mouse hair cells [RT plus (lane 1), RT minus (lane 2)]. **(C-H)** RNA *in situ* hybridization. Whole mount 4-dpf zebrafish treated with probes antisense to *svila* **(C)** and *svilc* **(F)** mRNAs. Both genes are expressed in the otocyst (arrowheads). Controls are displayed. Magnified otocysts show *svila* **(D)** and *svilc* **(G)** are expressed in the anterior macula (AM). Sense-probed controls **(E,H)**. White dashed lines denote the otic vesicles. Yellow boxes show positions of AM hair cells. PC indicates the region of the posterior cristae (out of focus).

To determine whether supervillin family members are expressed in hair cells of other vertebrates, we conducted RT-PCR using RNA from isolated zebrafish and mouse hair cells. Zebrafish have two or more paralogs for many mouse and human genes due to a whole genome duplication event in an evolutionary ancestor of teleost fishes [36]. A search of the zebrafish genome resulted in identification of four zebrafish supervillin genes, *svila* on chromosome 12 (XM_009306580.1, 56.94% identity with human *SVIL* [XM_011519633.1] by alignment using Clustal Omega), *svilb* on chromosome 2 (XM_009298867.1, 53.22% identity with human), *svilc* on chromosome 3 (XM_001344915.5, 41.14% identity with human), and *svild* on chromosome 12 (XM_009306541.1, 43.16% identity with human). *Svild* and *svila* are on the same chromosome but separated by over 10 million base pairs, suggesting they are distinct genes. Expression of *svila* and *svilc* gene products were detected in zebrafish hair cell cDNA using intragenic primer sets (Fig 2A). We were unable to detect expression of *svilb* and *svild* in zebrafish hair cells. However, expression of these genes was detected in zebrafish whole maculae (Fig 2A), indicating they may not be expressed in hair cells or are expressed at much lower levels. *Svil* gene expression was also detected in mouse hair cell cDNA (Fig 2B). To confirm expression of *svila* and *svilc* in the zebrafish ear, we performed mRNA *in situ* hybridization on 4-days post-fertilization (dpf) embryos. Both *svila* and *svilc* were detected in the ear at the anterior macula (Fig 2C–2H) as well as the posterior macula and cristae (data not shown).

### Supervillin localizes to the CP in mouse vestibular and cochlear hair cells and to apicolateral margins of cochlear supporting cells

**Fig 3:**
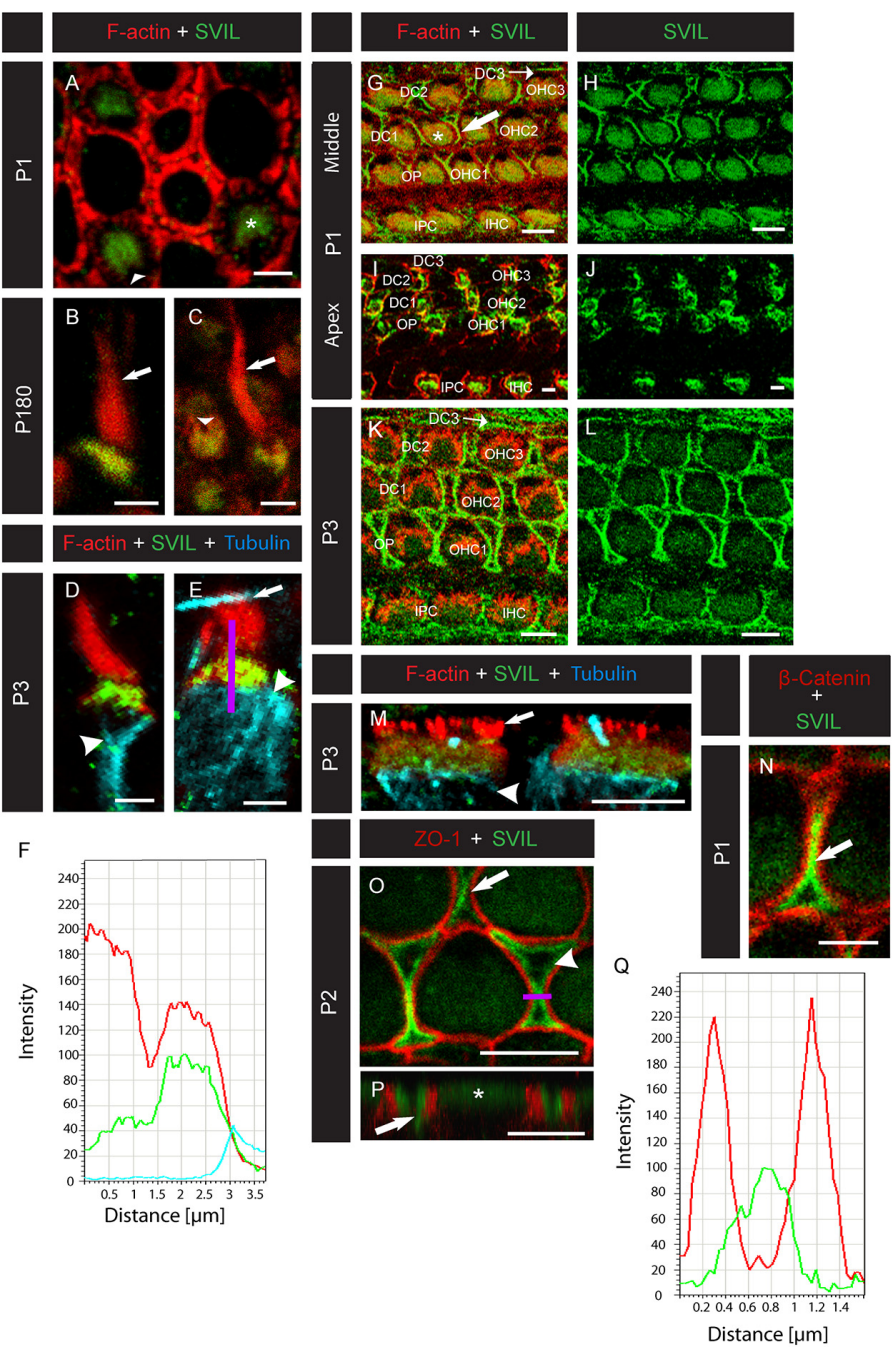
Supervillin localizes to mouse hair cell CPs and cochlear supporting cell head plates. **(A-E)** Confocal micrographs of mouse vestibular hair cells labeled with anti-SVIL (green) and phalloidin (red) at different developmental stages. (**A**) A top-down view of several hair cells from a mouse at P1. Supervillin labels the CPs (asterisk) but not the fonticulus (arrowhead). **(B,C)** Views of hair cells from a 6-month-old mouse. Supervillin labels the CP but not the fonticulus (arrowhead) or stereocilia (arrows). (**D,E**) Type I (**D**) and type II (**E**) vestibular hair cells from a P3 mouse co-labeled with anti-tubulin (blue), which marks somatic microtubules underlying the CPs (arrowheads). Arrow in (**E**) indicates a kinocilium from a neighboring hair cell. A region of interest (ROI, indicated by the purple line) was selected to span the hair bundle (top portion of the line), CP (middle portion of the line), and underlying microtubules (bottom portion of the line). (**F**) Fluorescence intensity profile using the ROI from **(E)**. The hair bundle (top portion of the purple ROI line) corresponds to the left region of the plot, showing robust F-actin-associated signal (red), while SVIL- (green) and tubulin-associated (blue) signals are minimal. The middle region of the plot corresponds to the CP (middle portion of the ROI line) and shows overlapping SVIL- and F-actin-associated signals; however, in the right region of the plot, only tubulin-associated signal is seen below the CP (bottom portion of the ROI line). (**G-M**) Confocal micrographs of mouse cochlear hair cells labeled with anti-SVIL (green) and anti-actin (red). (**G-J**) Hair cells at the middle (**G,H**) and apical (**I,J**) cochlear turns of a P1 mouse. At the middle turn (**G,H**), SVIL localizes to the CPs (asterisk) and to the region of the hair cell-supporting cell junctions (arrow). At the apical turn (**I,J**), SVIL co-localizes with actin near the apical surface of the developing hair cells. (**K,L**) In the middle turn of the P3 mouse cochlea, SVIL localizes to CPs of outer hair cells (OHCs) and inner hair cells (IHCs) and to supporting cell apicolateral margins, including those of Deiters’ cells (DC1, DC2, DC3), outer pillar cells (OP), and inner phalangeal cells (IPC). (**M**) Side view of two IHCs from the middle turn of a P3 mouse cochlea co-labeled with an antibody to tubulin (blue) demonstrates that SVIL localizes between the hair bundle (arrow) and somatic microtubules (arrowhead), at the region of the cuticular plate (asterisk). (**N**) Magnification of two OHCs and the Deiters’ cell between them (arrow) from the basal turn of a P1 mouse cochlea labeled with anti-SVIL (green) and anti-β-catenin (red). (**O**) Magnification of the first two rows of OHCs from the basal turn of a P2 mouse cochlea labeled with anti-SVIL (green) and anti-ZO-1 (red). SVIL strongly localizes to the apicolateral margins of the OPs (arrowhead) and the DCs (arrow). (**P**) Z-stacks of confocal sections were converted into a 3D model using the Leica Software. A 3D reconstruction of an OHC (asterisk) from the second row flanked by two DCs (arrow) labeled with anti-SVIL (green) and anti-ZO-1 (green) is seen. (**Q**) Fluorescence intensity profile of the cell in (**O**) using the ROI indicated by the purple line demonstrates that the signal associated with SVIL (green) is concentrated in the supporting cells, sandwiched between ZO-1-rich bands (red). In graphs in **F** and **Q**, intensity scales are linear, but the units are arbitrary. Scale bars, 2 μm.

To determine whether supervillin could be involved in shaping F-actin structures near the apical surface of hair cells, we examined localization of supervillin protein in the murine ear by immunolabeling. Specificity of the H340 antibody used to detect mouse SVIL has been successfully demonstrated previously [31, 37]. In methanol-fixed mouse vestibular hair cells, supervillin localizes to the CP at both postnatal day 1 (P1) (Fig 3A) and adult stages (Fig 3B and 3C). Co-labeling with phalloidin and an antibody to tubulin, which labels the somatic microtubules underlying the CP, showed that SVIL localizes to the region between the hair bundle and the somatic microtubules, at the region of the CP (Fig 3D–3F). This labeling pattern was consistent in both type I (Fig 3D) and type II (Fig 3E) vestibular hair cells. In the mouse cochlea middle turn at P1 and P3, supervillin localizes to the CP and near the apical junctional complexes of supporting cells that surround hair cells (Fig 3G, 3H, 3K and 3L), including the head plates of Deiters’ and outer pillar cells and the apicolateral margins of inner phalangeal cells [10, 15, 38]. SVIL also co-localizes with F-actin near the apical surface of developing hair cells at the apical turn of the cochlea at P1 (Fig 3I and 3J). CP labeling is generally stronger at P1 (Fig 3G and 3H) and weakens by P3 (Fig 3K and 3L), though there was some variability from sample to sample. This may be due to thickening of the CP as hair cells develop [39], which may obscure access by the SVIL antibody. Alternatively, the apical epithelia of the organ of Corti undergoes re-shaping during this time [40], and supervillin may be replaced by another cytoskeletal protein. As with the vestibular hair cells, co-labeling of cochlear hair cells with anti-SVIL and anti-tubulin showed that SVIL labels the CP, between the somatic microtubules and the hair bundle (Fig 3M). Some supervillin-associated signal was occasionally detected in the hair bundle; however, this signal was not consistent and did not seem to follow any specific pattern.

The localization of supervillin near the apicolateral margins of supporting cells is interesting, as large circumferential belts of F-actin and myosin II are found at these regions, associated with tight and adherens junctions with neighboring hair cells [3, 10, 41]. Myosin IIA, myosin IIB, and myosin IIC heavy chain proteins are all expressed during cochlear morphogenesis [42], and myosin IIB heavy chain localizes near the cellular junctions in the developing cochlea [41]. Supervillin interacts with both myosin IIA and myosin IIB heavy chains [23]. Interestingly, the genes encoding myosin IIA (*MYH9*) and myosin IIC (*MYH14*) heavy chains are associated with DFNA17 and DFNA4 nonsyndromic hearing loss, respectively [43, 44]. To further investigate the precise localization of supervillin in supporting cells, we co-labeled P1 mouse cochleae using antibodies recognizing supervillin and adherens junction marker β-catenin and tight junction marker ZO-1. Supervillin localizes adjacent to β-catenin and ZO-1, and is concentrated on the supporting cell side of hair cell-supporting cell junctions (Fig 3N–3Q). From these data, we propose supervillin is involved in organizing cytoskeletal structure at the circumferential belts in supporting cells, including those of the head plates; this hypothesis is supported by established biochemical properties of supervillin [19, 20]. In adult mice, the circumferential F-actin belts in supporting cells are wider than those of hair cells [45, 46]. In cultured epithelial cells, supervillin promotes myosin II contractility and recruitment of F-actin into bundles proximal to the plasma membrane [20]. Thus, supervillin may be involved in the development of the specialized wider circumferential belts in supporting cells, but may not be needed for the development of the comparatively thinner circumferential belts in the hair cells. These findings imply that supervillin is involved in not only CP structure, but also in establishing the global integrity of the reticular lamina. Interestingly, vestibular supporting cells did not express supervillin (Fig 3A and 3C), indicating that either the extra structural support established by supervillin is not necessary for the vestibular epithelia, or a different protein plays this role in the vestibular system. This may reflect unique stresses imposed on the reticular lamina by auditory stimuli [47, 48].

### Supervillin localizes to the zebrafish hair cell CP

To resolve whether supervillin also localizes to the CP of non-mammalian hair cells, and is therefore evolutionarily conserved, we generated novel polyclonal antiserum recognizing a 321-amino acid, N-terminal fragment of zebrafish Svila (Fig 1B). Specificity of our antibody was demonstrated by immunolabeling in zebrafish injected with a morpholino targeting the translation start site of Svila. The fluorescence intensity of anti-Svila labeling at the CP of 4-dpf fish was compared to that of anti-acetylated tubulin labeling of the underlying microtubules. The 5-bp mismatch control-injected fish had an anti-Svila labeling fluorescence intensity that was 2.48 ± 0.29 (ratio of means in arbitrary fluorescence units ± standard error of the mean) (n = 20 hair cells from 4 fish) times as strong as that of anti-tubulin (S2B Fig). The relative Svila fluorescence intensity was greatly diminished in the morphant fish in which the anti-Svila fluorescence intensity was only 0.65 ± 0.07 (n = 20 hair cells from 4 fish) times that of anti-tubulin (S2A Fig). The fluorescence intensity of anti-acetylated tubulin labeling was very similar between the morphant and control fish (average arbitrary fluorescence units = 90.2 ± 43 in morphant fish and 90.2 ± 69 in control fish; n = 20 hair cells from 4 fish in each group), indicating that the amount of tubulin in the hair cells was not affected by the morpholino. The morphant fish developed grossly normal cuticular plates (S2C and S2D Fig). However, some Svila protein was still detected in the CPs of the morphant fish (S2A Fig), indicating that the knockdown was incomplete, but successfully demonstrating the specificity of the antibody.

**Fig 4:**
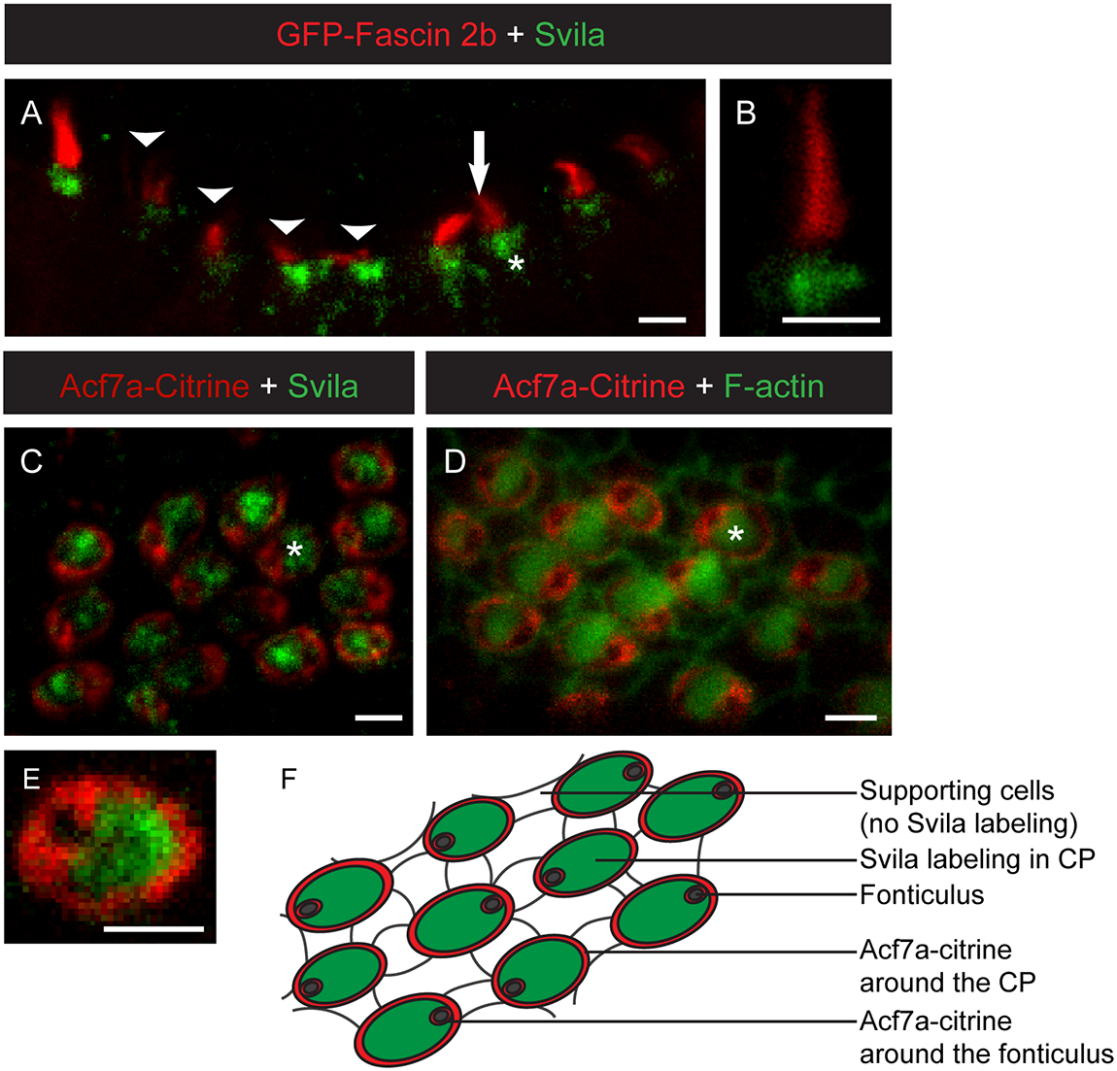
Supervillin localizes to zebrafish hair cell CPs. **(A,B)** Confocal micrographs of 4-dpf zebrafish anterior crista hair cells expressing GFP-fascin 2b (red) and labeled with anti-Svila (green). Arrowheads in **A** indicate hair cells out of focus or bent. Arrow indicates hair-bundle-localized GFP-fascin 2b. Asterisk indicates a CP. **(C,D,E)** Confocal micrographs of posterior macula hair cells from zebrafish expressing Acf7a-Citrine (red) and labeled with anti-Svila (green) **(C,E)** or phalloidin (green) **(D)**. Asterisks in **(C,D)** indicate CPs. Acf7a-Citrine encircles the CP, localizes to the CP base, out of the focal plane in **C-E**, and is found weakly throughout the CP. (**F**) Schematic of the zebrafish posterior macula tissue with the location of Svila immunolabeling in green and Acf7a-Citrine indicated in red. Scale bars, 2 μm.

Fixation conditions necessary for successful Svila immunolabeling with our new antiserum were incompatible with F-actin labeling. Thus, to determine localization of Svila relative to the hair bundle, we carried out immunolabeling in GFP-fascin 2b transgenic fish, which maintain correct localization of fluorescently-tagged hair bundle-specific protein fascin 2b [25, 26]. In 4-dpf embryos, Svila localizes immediately below the bundle, at the location of the CP (Fig 4A and 4B) but was not detected in the supporting cells. Localization of Svila to the CP is further confirmed by immunolabeling in *Gt(macf1a–citrine)*^*ct68a*/+^ zebrafish expressing Acf7a-Citrine fusion protein [24] (Fig 4C–4E), which circumscribes the CP in higher concentrations and is enmeshed in the CP in lower concentrations [27]. Svila localizes throughout the region circumscribed by Acf7a-Citrine (Fig 4C, 4E and 4F). We therefore conclude supervillin localizes to the CP of zebrafish hair cells.

**Fig 5:**
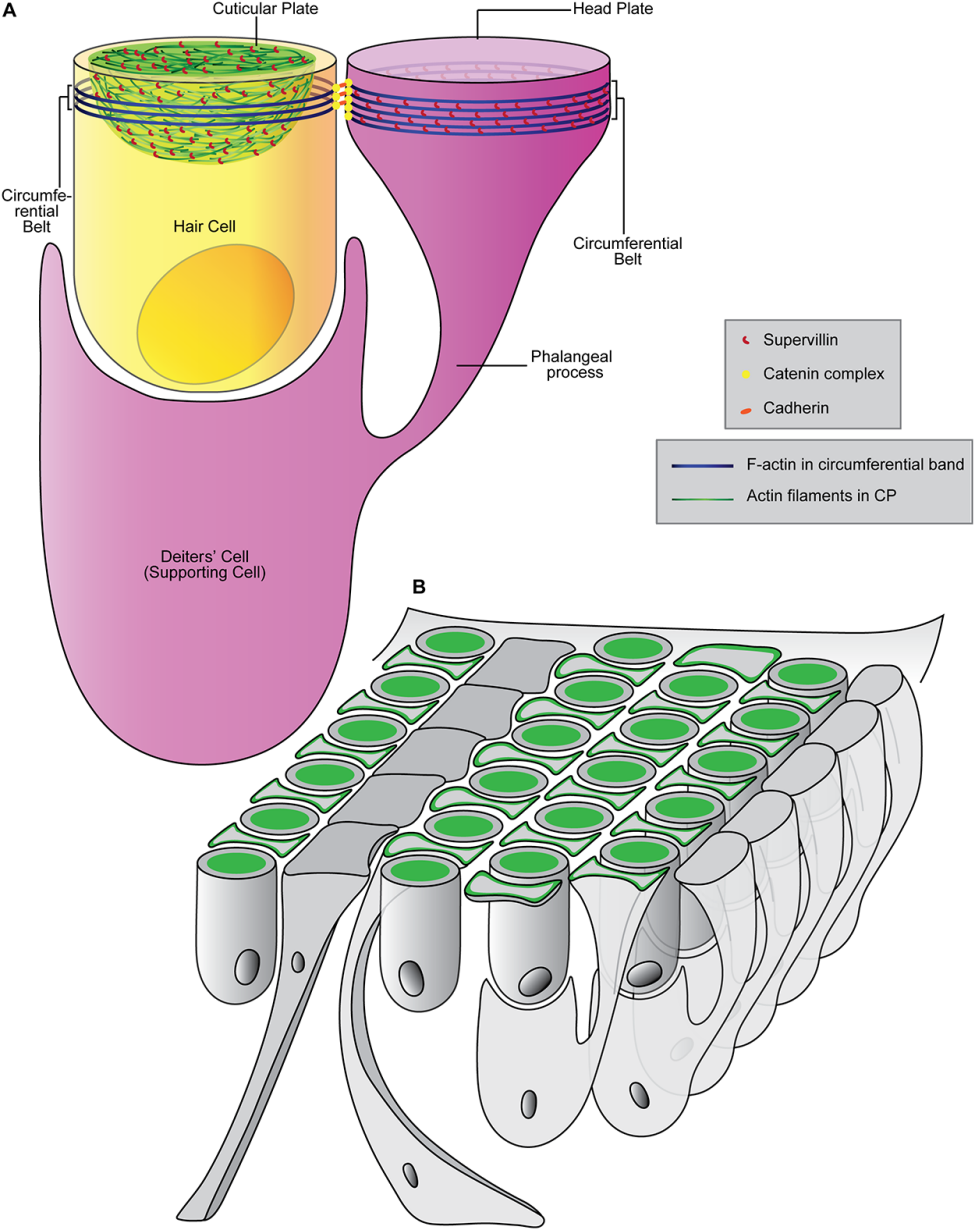
Models of supervillin function. **(A)** Model of supervillin (red) as an F-actin-organizing protein in the hair cell CP (green) and the circumferential belt (blue) of a Deiters’ cell. Hair bundle not shown. (**B**) Schematic of supervillin localization (green) in the organ of Corti. Supervillin is found in CPs of IHCs and OHCs, the apicolateral margins of the head plates of DCs and OPs, and apicolateral margins of IPCs.

Based on its localization in mouse and zebrafish hair cells and its known cytoskeletal functions [19, 20], we propose that supervillin contributes to CP shape and integrity (Fig 5A). Also of interest is localization of supervillin near the developing cellular junctions of supporting cells in the organ of Corti, where supervillin may serve to organize actin filaments and myosins (Fig 5A and 5B). The fact that supervillin was not detected in inner pillar cells may reflect the unique shape of their head plate, which extends to cover part of the process of the neighboring outer pillar cell [11]. It is thus possible that different molecular mechanisms are involved in shaping the F-actin belts of the inner pillar cells. Further genetic studies of supervillin may advance our understanding of the development of the poorly understood CP and the specialized apicolateral regions of Deiters’, outer pillar, and inner phalangeal cells, which define the intricate architecture of the organ of Corti.

## Supporting Information

S1 Fig Schematic of the mammalian organ of Corti. The organ of Corti contains three rows of outer hair cells (OHCs) and one row of inner hair cells (IHCs). OHCs are flanked by Deiters’ cells (DCs) and outer pillar cells (OPs), and IHCs are bordered by inner phalangeal cells (IPCs). Inner pillar cells (IPs) are between the OHCs and IHCs.
(TIF)

S2 Fig Morpholino knockdown of Svila in zebrafish. Confocal micrographs of 4-dpf zebrafish hair cells injected with a morpholino targeting Svila (**A,C**) or a 5-bp mismatch control morpholino (**B,D**). (**A,B**) Hair cells from the anterior macula labeled with anti-Svila (green) and anti-acetylated tubulin (red) reveal that the intensity of Svila protein at the cuticular plate (arrows) is diminished in Svila morpholino-injected fish (**A**) compared to fish injected with control (**B**), but some Svila protein is still detected (**A**). Fluorescence intensity of anti-Svila at the CP was compared to that associated with anti-tubulin labeling of the underlying microtubules (asterisks). Phalloidin labeling of neuromast hair cells from Svila morpholino-injected (**C**) and control-injected (**D**) fish reveals normal gross cuticular plate structure in the morphants.
(TIF)

S1 Table Primers used to amplify cDNA of *supervillin* genes. (TIF)
