# Correction: Supervillin is a Component of the Hair Cell's Cuticular Plate and the Head Plates of Organ of Corti Supporting Cells

**DOI:** 10.1371/journal.pone.0161638

**Published:** 2016-08-17

**Authors:** The PLOS ONE Staff

The following information is missing from the Funding section of the published article: E.J.L. was funded by NIH grant GM033048-26S1. The publisher apologizes for the error.
