# Supplementary figures and images for: Supervillin Is a Component of the Hair Cell’s Cuticular Plate and the Head Plates of Organ of Corti Supporting Cells
Source: PLoS One. 2016 Jul 14;11(7):e0158349. doi: 10.1371/journal.pone.0158349 (PMC4944918; doi:10.1371/journal.pone.0158349)

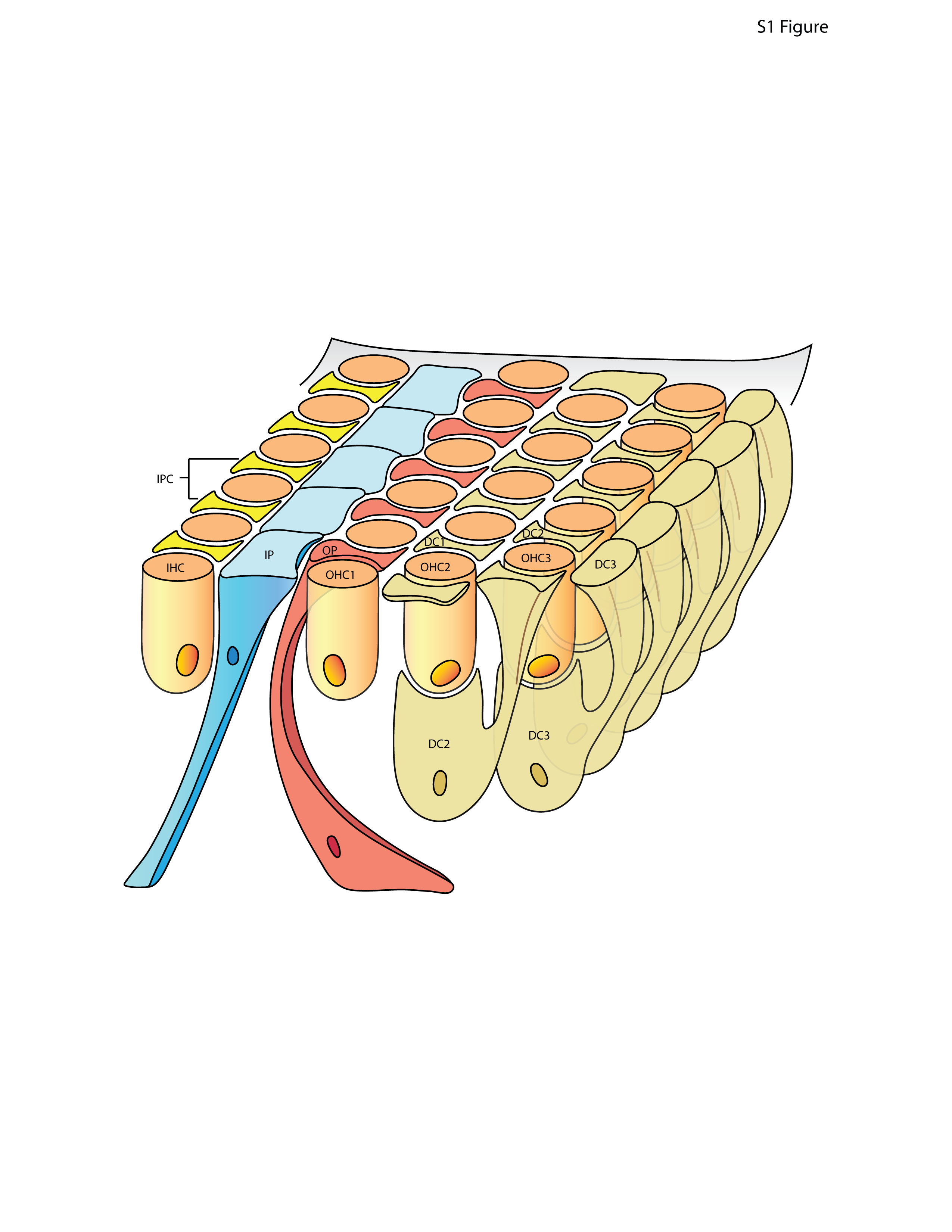

Supplement: S1 Fig — The organ of Corti contains three rows of outer hair cells (OHCs) and one row of inner hair cells (IHCs). OHCs are flanked by Deiters’ cells (DCs) and outer pillar cells (OPs), and IHCs are bordered by inner phalangeal cells (IPCs). Inner pillar cells (IPs) are between the OHCs and IHCs. (TIF) [file pone.0158349.s001.tif]

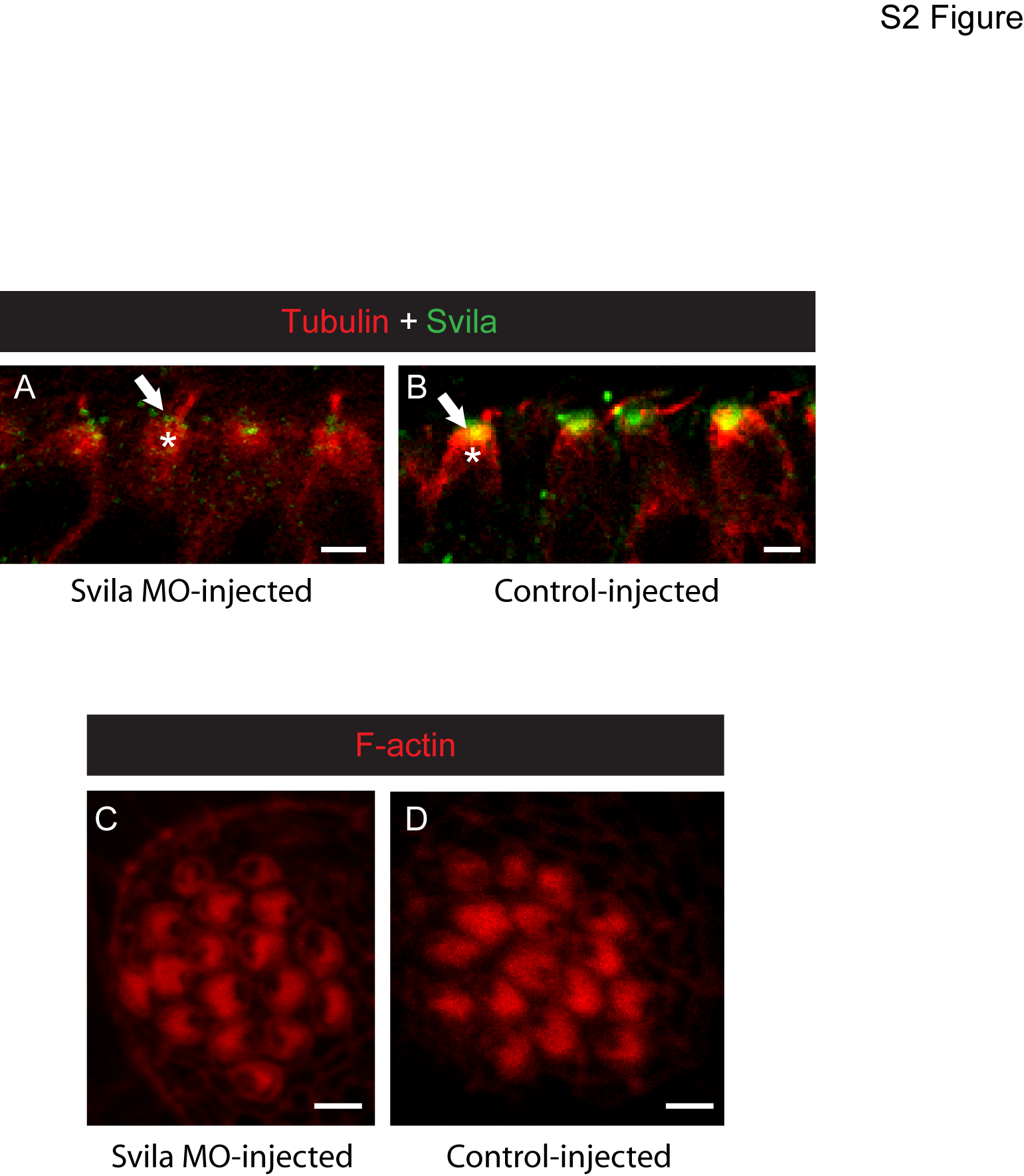

Supplement: S2 Fig — Confocal micrographs of 4-dpf zebrafish hair cells injected with a morpholino targeting Svila (A,C) or a 5-bp mismatch control morpholino (B,D). (A,B) Hair cells from the anterior macula labeled with anti-Svila (green) and anti-acetylated tubulin (red) reveal that the intensity of Svila protein at the cuticular plate (arrows) is diminished in Svila morpholino-injected fish (A) compared to fish injected with control (B), but some Svila protein is still detected (A). Fluorescence intensity of anti-Svila at the CP was compared to that associated with anti-tubulin labeling of the underlying microtubules (asterisks). Phalloidin labeling of neuromast hair cells from Svila morpholino-injected (C) and control-injected (D) fish reveals normal gross cuticular plate structure in the morphants. (TIF) [file pone.0158349.s002.tif]

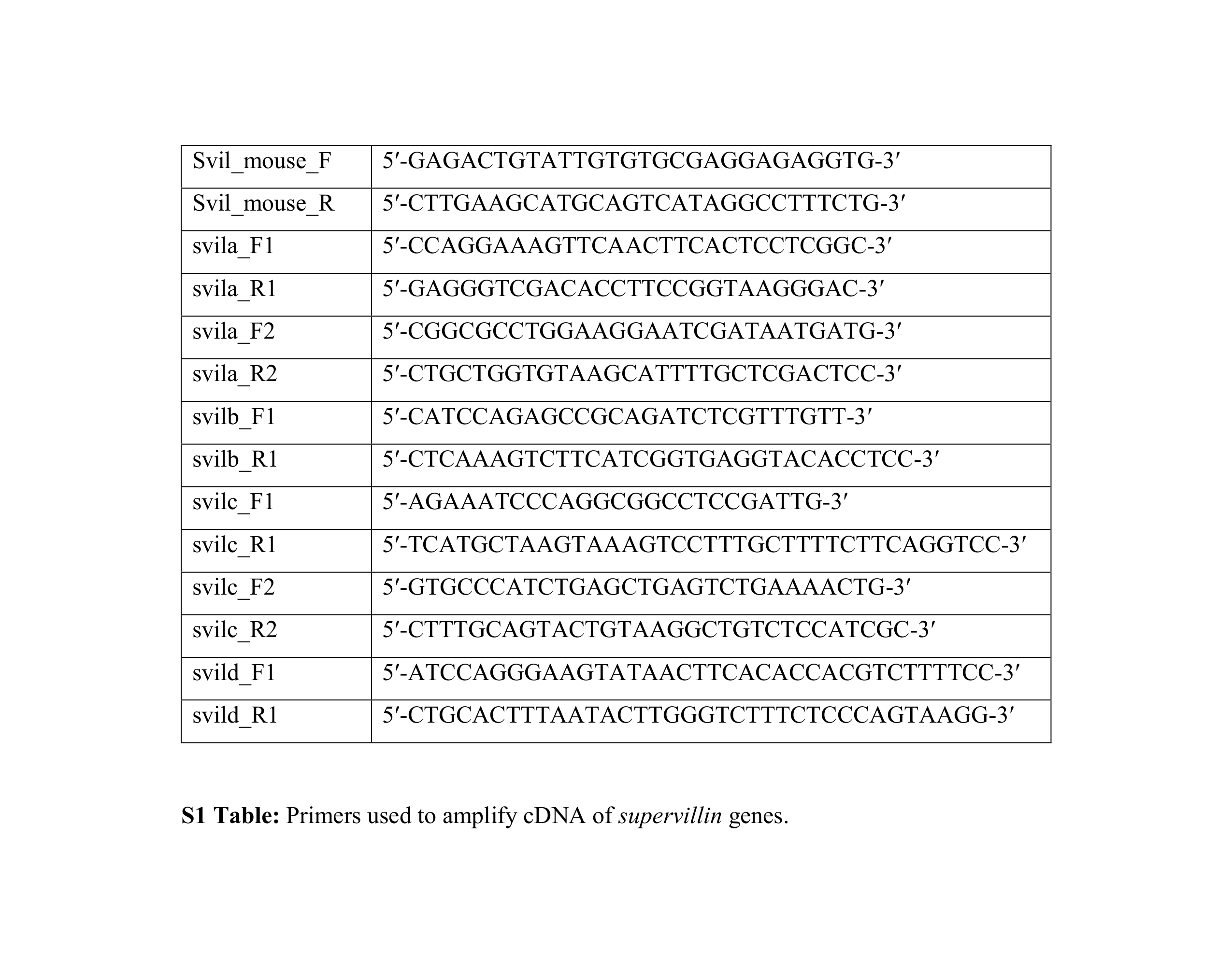

Supplement: S1 Table — (TIF) [file pone.0158349.s003.tif]
